# Treatment efficacy of first-line immunotherapy in advanced esophageal small cell carcinoma: A real-world retrospective study

**DOI:** 10.3389/fonc.2025.1630210

**Published:** 2025-10-02

**Authors:** Nan Li, Rui xing Zhang

**Affiliations:** Department of Gastroenterology, Fourth Hospital of Hebei Medical University, Shijiazhuang, China

**Keywords:** esophageal small cell carcinoma, immune checkpoint inhibitors, therapeutic efficacy, adverse event, prognostic analysis

## Abstract

**Background:**

Small cell carcinoma of the esophagus (SCCE) is a rare and highly aggressive malignancy characterized by early metastatic propensity. Traditional chemotherapy has a poor curative effect and a short survival period Recent evidence demonstrates that combining immune checkpoint inhibitors (ICIs) with chemotherapy significantly improves therapeutic outcomes in both advanced esophageal carcinoma and small cell lung cancer. This study aims to evaluate the efficacy of first-line ICIs combined with chemotherapy in patients with advanced SCCE.

**Patients and methods:**

This single-center retrospective study analyzed 31 patients with advanced SCCE who initiated first-line systemic therapy at our institution between January 2021 and August 2024. All patients received physician-determined treatment regimens. The study aimed to evaluate the efficacy of first-line immunotherapy combined with chemotherapy in advanced SCCE.

**Results:**

Median progression-free survival (PFS) was significantly longer in the immunotherapy group (9.3 months; 95% CI 6.3-12.3) compared to the non-immunotherapy group (5.4 months; 95% CI 3.5-7.3; *P* = 0.046). Patients receiving chemotherapy alone demonstrated the shortest PFS (3.2 months; 95% CI 2.1-4.3), while those receiving combined chemotherapy and immunotherapy achieved the longest PFS (10.0 months; 95% CI 3.8-16.1). Median overall survival (OS) of patients with combined immunotherapy showed a trend of prolongation (17.0 months 95% CI 12.9-21.13 *vs*. 11.6 months 95% CI 4.7-18.6), but no statistically significant difference was observed (p = 0.055). Multivariate analyses suggested that the combination of immunotherapy, or its absence, may affect patient prognosis. Numerical improvements were observed in the immunotherapy group for both objective response rate (ORR: 31.3% *vs*. 21.4%) and disease control rate (DCR: 93.7% *vs*. 85.7%).

**Conclusion:**

Esophageal small cell carcinoma remains a highly aggressive malignancy with poor prognosis in advanced stages. This retrospective real-world study suggests that first-line immunotherapy combined with chemotherapy may significantly improve progression-free survival in patients with advanced SCCE compared to chemotherapy alone.

## 1 Introduction

Small cell carcinoma of the esophagus (SCCE) is a lethal malignancy with a dismal prognosis, accounting for 0.8%-2.4% of all esophageal cancers. It exhibits high aggressiveness and an early propensity for metastasis. Although its biological behavior resembles that of small cell lung cancer (SCLC), its prognosis is notably worse. The 5-year survival rate for SCCE is less than 5%, and only 10% of patients survive longer than one year (1).

For advanced SCCE, multidrug chemotherapy (typically including cisplatin or carboplatin with etoposide) remains the primary treatment. While radiotherapy may enhance local control, recurrence and drug resistance rates are high (2). Recently, the combination of immune checkpoint inhibitors (ICIs) with chemotherapy has become the standard first-line therapy for esophageal squamous cell carcinoma (ESCC), offering potential synergistic benefits and significantly improved efficacy in the advanced setting (3). Similarly, in first-line treatment of SCLC, combining ICIs (such as atezolizumab or durvalumab) with chemotherapy has demonstrated significant survival advantages over chemotherapy alone (4, 5). Given this background, we conducted a retrospective analysis of SCCE patients at our center to evaluate whether combining chemotherapy with immunotherapy improves survival outcomes in SCCE.

## 2 Methods

### 2.1 Study design and participants

This retrospective study collected data from SCCE patients treated at the Fourth Hospital of Hebei Medical University between January 2021 and August 2024. Inclusion criteria were: (1) histologically confirmed, previously untreated SCCE;.Exclusion criteria included: (1) history of malignancy; (2) clinical stage T1N0M0 or earlier; and (3) incomplete information or loss to follow-up. The study protocol was approved by the institutional review board of our institution. Due to the retrospective nature of this study, the ethics committee waived informed consent.

All patients had a histologically confirmed SCCE diagnosis based on immunohistochemical staining for common neuroendocrine markers, including neuron-specific enolase (NSE), synaptophysin (Syn), chromogranin A (CgA), cytokeratin 56 (CK56), cytokeratin (CK), and lymphocyte antigen 56 (CD56). Staging was performed using both the 8th edition of the UICC/AJCC TNM Classification of Carcinoma of the Esophagus and Esophagogastric Junction and the Veterans Administration Lung Study Group (VALSG) staging system.

Routine baseline examinations included physical examination, endoscopy with biopsy, and contrast-enhanced computed tomography (CT).

### 2.2 Treatment

Due to the absence of standardized treatment guidelines, the therapeutic strategy was determined by physician discretion and patient preference. Chemotherapy regimens primarily consisted of platinum-based agents (e.g., cisplatin or carboplatin) combined with other cytotoxic drugs such as etoposide or paclitaxel. Administered immune checkpoint inhibitors (ICIs) comprised Sintilimab, Serplulimab, and Adebelimab. Radiation oncologists planned radiotherapy based on tumor location, size, and involvement of regional lymph nodes, delivering doses ranging from 45 Gy to 55 Gy.

### 2.3 Statistical analysis

Clinicopathologic characteristics of patients and progression-free survival (PFS) and overall survival (OS) were compared between treatment groups. Additionally, objective response rate (ORR) and disease control rate (DCR) were compared. Categorical variables are presented as frequencies and percentages, and comparisons between groups were performed using the χ² test. PFS and OS were estimated using the Kaplan-Meier method and compared using the log-rank test. Hazard ratios (HRs) and their corresponding 95% confidence intervals (CIs) were calculated using Cox proportional hazards regression models. P<0.05 was considered statistically significant.

## 3 Results

### 3.1 Patient characteristics and treatment

A total of 39 patients were enrolled in the trial. Eight cases were excluded for not meeting the inclusion criteria: one patient staged as T1N0M0, two patients with concurrent other tumors, two patients who received neoadjuvant chemotherapy, two patients who did not receive chemotherapy, and one patient lost to follow-up. Consequently, 31 patients comprised the per-protocol population.

The cohort exhibited a male predominance. Tumors arose primarily in the mid-esophagus (45%) and lower esophagus (45%). The liver (26%) and distant lymph nodes (23%) were the most common metastatic sites.

Patients were divided into two treatment arms: Arm A (n=15) received chemotherapy and/or radiotherapy, while Arm B (n=16) received chemotherapy and/or radiotherapy plus immune checkpoint inhibitors. Demographic and clinical characteristics were generally comparable between the two arms, except for age (**Table 1**).

**Table 1:** Clinical Characteristics of the Study Patients.

Patient characteristic	A(n=15)	B(n=16)	*P*
Gender			0.39
Men,N(%)	11(73)	14(87)	
Women,N(%)	4(27)	2(13)	
Age Median ,(IQR)	66(59-72)	73(68-78)	0.02
Smoking,N(%)	8(53)	12(75)	0.28
Drinking,N(%)	4(26.7)	4(25)	0.92
Hypertension,N(%)	7(46.7)	10(63)	0.38
Primary tumor location, N(%)			0.86
Upper esophagus,N(%)	1(7)	2(13)	
Midesophageal,N(%)	7(47)	7(44)	
Lower esophagus,N(%)	7(47)	7(44)	
Clinical T stage,N(%)			0.26
T1	0(0)	1(6)	
T2	1(7)	0(0)	
T3	12(80)	10(63)	
T4	1(7)	3(18)	
missing	1(7)	2(13)	
Clinical N stage,N(%)			0.76
N0	2(13)	2(13)	
N1	5(33)	5(31)	
N2	3(20)	5(31)	
N3	4(27)	2(13)	
missing	1(7)	2(13)	
Clinical M stage,N(%)			0.45
M0	8(53)	6(38)	
M1	6(40)	8(50)	
missing	1(7)	2(12)	
VALSG,N(%)			0.58
limited (LD)	9(60)	8(50)	
extensive disease (ED)	6(40)	8(50)	
Metastatic site,N(%)			
Liver	5(33)	3(19)	0.35
Lung	1(7)	2(13)	0.58
Distant lymph node	3(20)	4(25)	0.74
Number of metastatic sites,N(%)			0.71
0	9(60)	8(50)	
1	3(20)	6(38)	
2	1(7)	1(6)	
3	2(13)	1(6)	
Ki67,N(%)			0.42
<80%	0	2(13)	
80%-89%	8(53)	7(44)	
≥90%	5(34)	5(31)	
missing	2(13)	2(13)	

### 3.2 Progression-free survival

**Figure 1:**
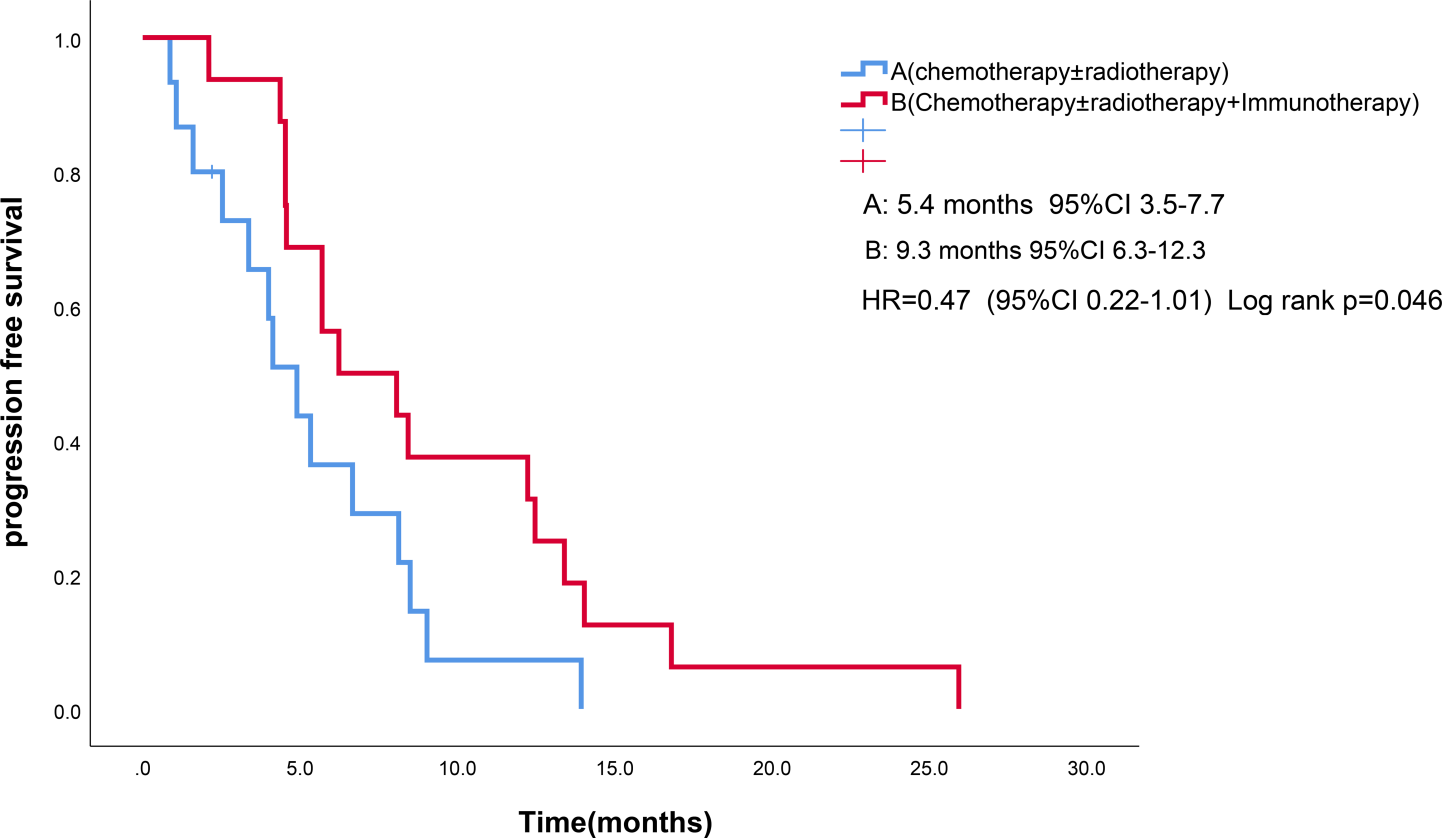
Kaplan-Meier survival estimates in PFS. **(A)**, chemotherapy and/or radiotherapy ; **(B)**, chemotherapy and/or radiotherapy plus ICIs; HR, hazard ratio; ICIs, Immune checkpoint inhibitors; PFS, Progression Free Survival.

**Figure 2:**
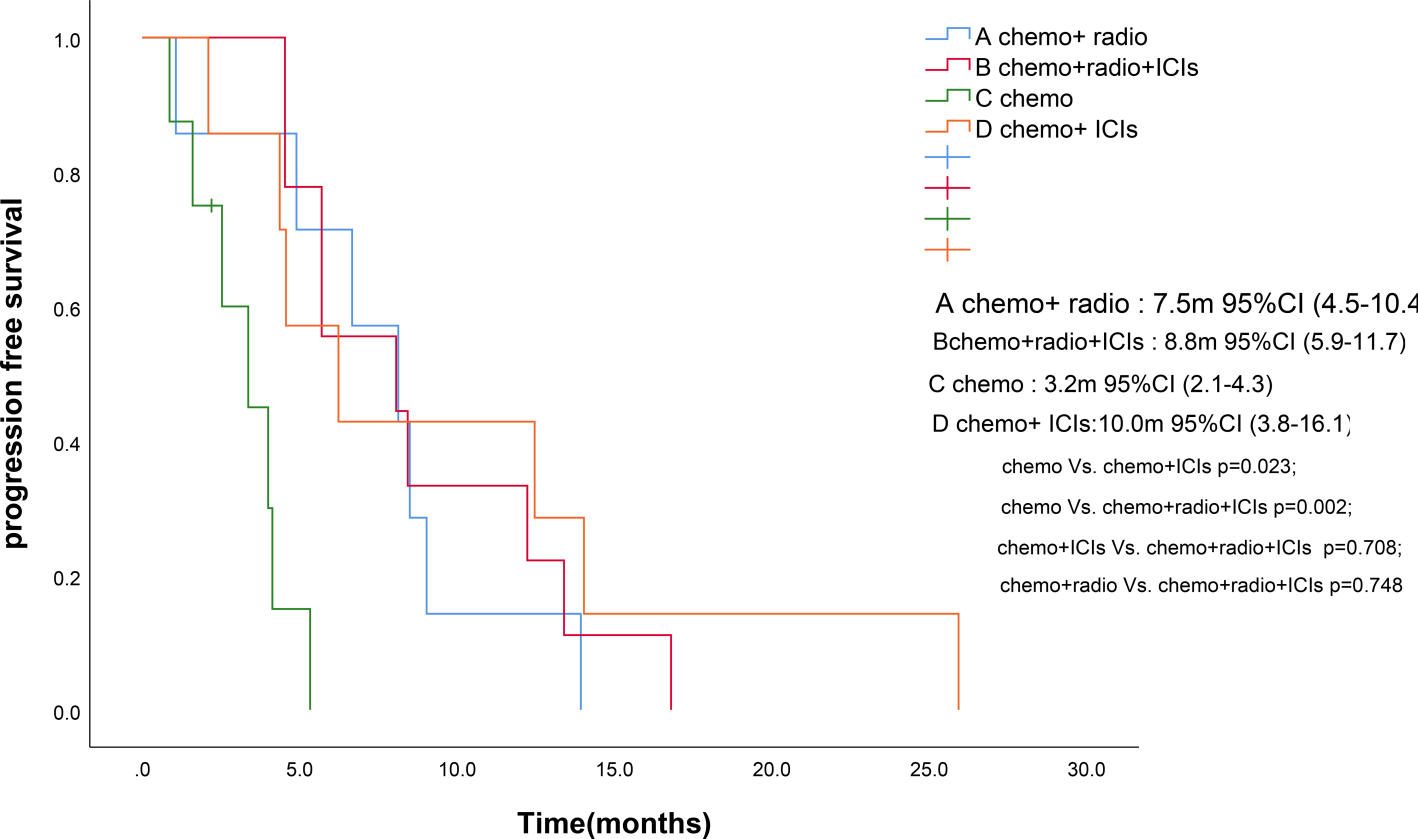
Kaplan-Meier survival estimates in PFS. **(A)**, chemotherapy and radiotherapy ; **(B)**, chemotherapy and radiotherapy and ICIs; **(C)**, chemotherapy; **(D)**, chemotherapy and ICIs. ICIs, Immune checkpoint inhibitors; PFS, Progression Free Survival. .

As of the follow-up cutoff in March 2025, one patient was lost to follow-up., PFS events were documented in 30 patients. The addition of ICIs to therapy significantly improved median PFS compared to the control group (9.3 months *vs*. 5.4 months, p = 0.046; **Figure 1**). Among the treatment subgroups, patients receiving chemotherapy alone exhibited the worst median PFS (3.2 months, 95% CI 2.1-4.3), while those receiving chemotherapy combined with ICIs demonstrated the best median PFS (10.0 months, 95% CI 3.8-16.1; **Figure 2**). Multifactorial analyses were performed to identify clinical features that might affect median PFS of patients (**Table 2**).

**Table 2:** Univariate and multifactorial analysis of risk factors for Progression Free Survival.

Variable	Univariable analysis		Multivariable analysis	
	OR (95% CI)	P	OR (95% CI)	P
Gender	Reference 2.545(0.391-16.550)	0.328	Reference 0.285(0.052-1.574)	0.150
Men				
Women				
Age(years)	Reference 0.861(0.761-0.975)	0.019	Reference 0.986(0.916-1.061)	0.710
Smoking	Reference 0.381(0.083-1.741)	0.213	Reference 1.846(0.600-5.676)	0.285
Drinking	Reference 1.091(0.218-5.454)	0.916		
Primary tumor location	Reference 0.500(0.036-6.862)	0.604		
Upper esophagus				
Midesophageal				
Lower esophagus				
Clinical T stage	Reference 0.282(0.026-3.113)	0.302	Reference 0.762(0.165-3.519)	0.728
T1-3				
T4				
Clinical N stage	Reference 1.500(0.211-10.649)	0.685		
N0				
N+				
Clinical M stage	Reference 1.778(0.398-7.943)	0.451		
M0				
M1				
VALSG	Reference 0.667(0.161-2.769)	0.577		
limited (LD)				
extensive disease (ED)				
Liver metastases	Reference 2.167(0.415-11.302)	0.359	Reference 0.653(0.222-1.926)	0.440
Lung metastases	Reference 0.500(0.410-6.166)	0.589		
Distant lymph node metastases	Reference 0.750(0.137-4.095)	0.740		
Number of metastatic sites	Reference 1.051(0.504-2.195)	0.894		
1				
2				
3				
PD-L1 CPS	Reference 0.958(0.819-1.220)	0.590		
Ki67	Reference 1.125(0.236-5.371)	0.883		
<90%				
≥90%				
NSE(ng/ml)	Reference 0.969(0.848-1.108)	0.646		
Treatment			Reference 0.311(0.110-0.873)	0.027
Chemotherapy±radiotherapy+Immunotherapy				
chemotherapy±radiotherapy				

### 3.3 Overall survival

**Figure 3:**
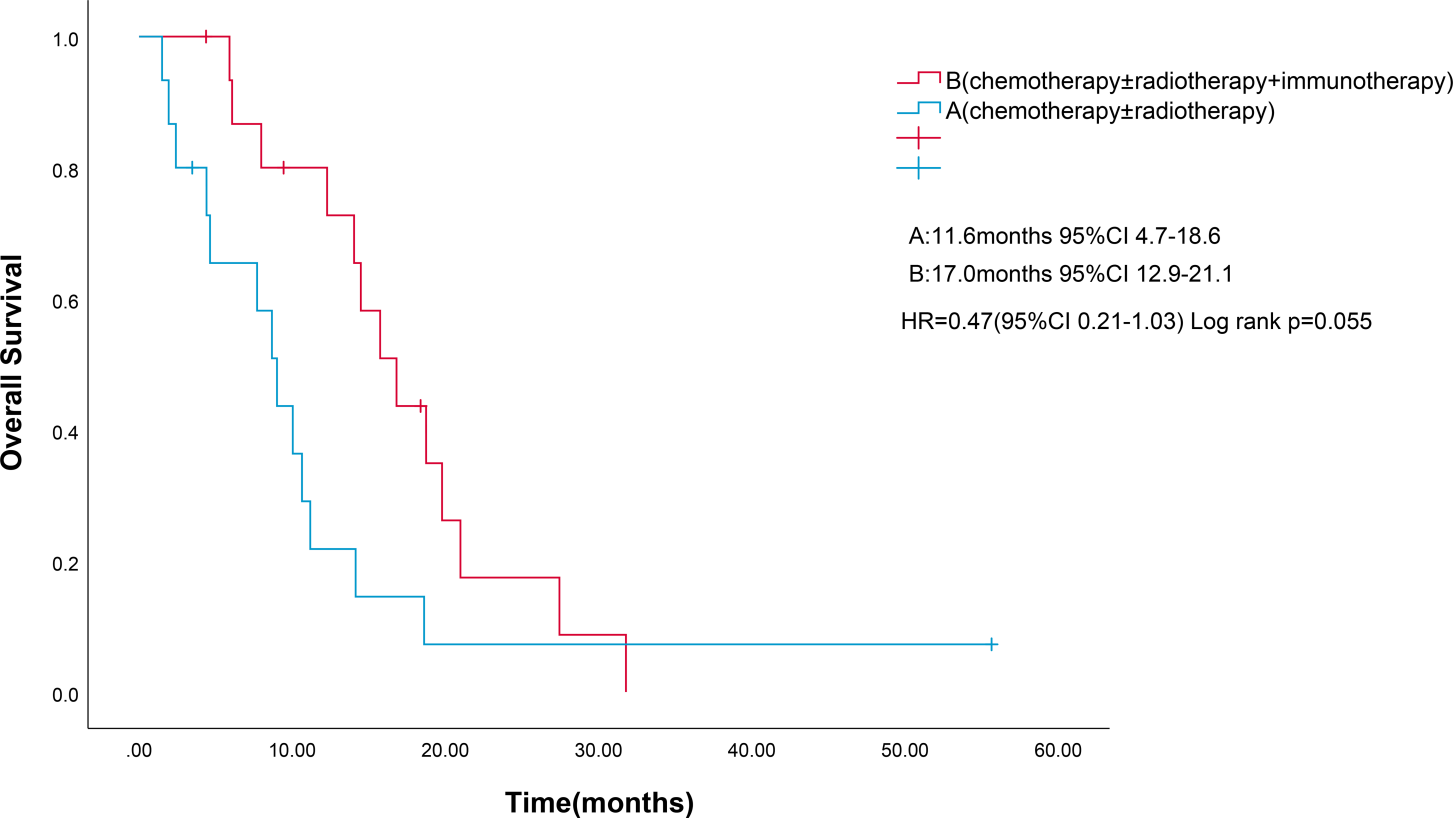
Kaplan-Meier survival estimates in OS. **(A)**, chemotherapy and/or radiotherapy; **(B)**, chemotherapy and/or radiotherapy plus ICIs; HR, hazard ratio; ICIs, Immune checkpoint inhibitors; OS, overall survival. .

Four patients were lost to follow-up for OS. OS events were documented in 27 patients. Median OS for combined immunotherapy and noncombined immunotherapy were 17.0 months(95% CI 12.9-21.13) and 11.6 months (95% CI 4.7-18.6), respectively (p=0.055) (**Figure 3**). Multifactorial analyses were performed to identify clinical features that might affect OS of patients (**Table 3**).

**Table 3:** Univariate and multifactorial analysis of risk factors for Overall Survival.

Variable	Univariable analysis		Multivariable analysis	
	OR (95% CI)	P	OR (95% CI)	P
Gender	Reference 2.545(0.391-16.550)	0.328	Reference 0.139(0.019-1.004)	0.050
Men				
Women				
Age(years)	Reference 0.861(0.761-0.975)	0.019	Reference 1.026(0.950-1.107)	0.515
Smoking	Reference 0.381(0.83-1.741)	0.213	Reference 1.506(0.394-5.762)	0.550
Drinking	Reference 1.091(0.218-5.454)	0.916		
Primary tumor location	Reference 1.231(0.414-3.684)	0.708		
Upper esophagus				
Midesophageal				
Lower esophagus				
Clinical T stage	Reference 3.545(0.321-39.136)	0.302	Reference 0.498(0.128-1.947)	0.316
T1-3				
T4				
Clinical N stage	Reference 1.500(0.211-10.649)	0.685		
N0				
N+				
Clinical M stage	Reference 0.563(0.126-2.513)	0.451		
M0				
M1				
VALSG	Reference 0.667(0.161-2.769)	0.577		
limited (LD)				
extensive disease (ED)				
Liver metastases				
Lung metastases	Reference 0.500(0.041-6.166)	0.589		
Distant lymph node metastases	Reference 0.750(0.137-4.095)	0.740		
Number of metastatic sites	Reference 1.051(0.504-2.195)	0.894		
1				
2				
3				
PD-L1 CPS	Reference 0.950(0.819-1.120)	0.590		
Ki67	Reference 0.889(0.186-4.244)	0.883		
<90%				
≥90%				
NSE(ng/ml)	Reference 0.969(0.848-1.108)	0.646		
Treatment			Reference 0.150(0.043-0.525)	0.003
Chemotherapy±radiotherapy+Immunotherapy				
chemotherapy±radiotherapy				

### 3.4 Efficacy

Treatment efficacy was evaluated in all patients. In Arm A, 3 patients (21.4%) achieved a partial response (PR), 9 patients (64.3%) had stable disease (SD), and 2 patients (14.3%) experienced progressive disease (PD). In Arm B, 5 patients (31.3%) achieved a PR, 10 patients (62.5%) had SD, and 1 patient (6.3%) experienced PD. The addition of ICIs was associated with a higher objective response rate (ORR: 31.3% *vs*. 21.4%) and disease control rate (DCR: 93.7% *vs*. 85.7%) compared to Arm A (**Table 4**).

**Table 4:** Efficacy comparison of the two groups.

Treatment efficacy assessment	B (chemotherapy±radiotherapy+immunotherapy)	A (chemotherapy±radiotherapy)	P
PR n(%)	5(31.3%)	3(21.4%)	0.38
SD n(%)	10(62.5%)	9(64.3%)	0.58
PD n(%)	1(6.3%)	2(14.3%)	0.27
ORR n(%)	5(31.3%)	3(21.4%)	0.38
DCR n(%)	15(93.7%)	12(85.7%)	0.27

### 3.5 Adverse events

The most common adverse events (AEs) were neutropenia (Arm A: 64.3% *vs* Arm B: 56.3%) and nausea (Arm A: 14.3% *vs* Arm B: 31.3%). The rate of grade 3–4 neutropenia was 42.9% in Arm A compared to 25.0% in Arm B. Compared with Arm A, the addition of ICIs was associated with an increase in grade 1–2 immune-related adverse events (irAEs), with an incidence rate of 25.0% (including 3 cases of thyroiditis and 1 case of colitis) (**Table 5**).

**Table 5:** Adverse reactions comparison of the two groups.

Treatment-related adverse events	Chemotherapy±radiotherapy+immunotherapy		Chemotherapy±radiotherapy	
	Allgrade	Grade≥3	Allgrade	Grade≥3
Neutropenia	9(56.3%)	4(25.0%)	9(64.3%)	6(42.9%)
Platelet count decreased	2(12.5%)	1(6.3%)	3(21.4%)	1(7.1%)
Nausea	5(31.3%)	0	2(14.3%)	0
Vomiting	3(18.8%)	0	1(7.1%)	0
Fatigue	4(25%)	1(6.3%)	3(21.4%)	0
Thyroiditis	3(18.8%)	0	0	0
Colitis	1(6.3%)	0	0	0

## 4 Discussion

SCCE is a rare malignant tumor characterized by low incidence but high malignancy. Its biological behavior resembles that of small cell lung cancer, yet it carries a worse prognosis.

SCCE occurs predominantly in males (6), exhibiting high proliferative activity and rich neovascularization (7). Tumors are commonly located in the middle or lower third of the esophagus (8). In our study, the liver (n=8, 26%) was the most common metastatic site, followed by distant lymph nodes, consistent with previous research (9).

The staging of SCCE is primarily based on the UICC/AJCC TNM staging system and the Veterans Administration Lung Study Group (VALSG) criteria. For patients with stage cT1-2N0M0 SCCE, adjuvant therapy significantly improved survival compared to surgery alone (5-year overall survival [OS]: 32.8% *vs*. 19.2%; median survival time [MST]: 44.0 *vs*. 33.0 months; *P* = 0.035) (10). In contrast, for advanced SCCE, no standardized treatment guidelines currently exist. Consequently, most patients receive chemotherapy or chemoradiotherapy. While multiple studies have demonstrated that chemoradiotherapy can improve survival rates in advanced SCCE, further clinical trials are needed to establish the optimal treatment approach (11).

Recently, immune checkpoint inhibitors (ICIs) have emerged as a promising treatment strategy for esophageal cancer. Previous studies demonstrated that ICIs can alleviate immunosuppressive effects and enhance anti-tumor activity (12). ICIs have shown significant survival benefits in patients with esophageal squamous cell carcinoma, with superior responses observed in those expressing higher levels of PD-L1 (3).

Currently, ICIs combined with chemotherapy represent the standard first-line treatment for advanced esophageal carcinoma. Supporting evidence comes from trials in small cell lung cancer (SCLC), a disease with biological similarities to SCCE. The CASPIAN trial established the efficacy of durvalumab combined with chemotherapy in treatment-naïve extensive-stage SCLC, demonstrating a significant improvement in median overall survival (13.0 months *vs*. 10.3 months) (4). Similarly, the IMpower133 trial showed that atezolizumab plus chemotherapy significantly improved overall survival (OS) and progression-free survival (PFS) in extensive-stage SCLC (5, 13).

Consistent with this trend, our study found that the addition of ICIs was associated with significant improvements in median PFS (9.3 *vs*. 5.4 months, P = 0.046), objective response rate (ORR: 31.3% *vs*. 21.4%), and disease control rate (DCR: 93.7% *vs*. 85.7%). In our study, median OS of patients with combined immunotherapy showed a trend of prolongation (17.0 months *vs*. 11.6 months), but no statistically significant difference was observed (p = 0.055). This may be due to the fact that some patients added immunotherapy to second-line treatment. Multivariate analyses suggested that the combination of immunotherapy, or its absence, may affect patient prognosis.

However, due to the limited sample size of this study, PD-L1, TMB, and MSI data could not be obtained, and the study involved a variety of immune checkpoint inhibitors, which may have caused some deviation in the results. The small sample size (n=31) and the unbalanced group sizes (15 versus 16) in this study may cause some deviation in the results. Larger studies are warranted to confirm the efficacy of ICIs specifically in SCCE, perform subgroup analyses, and identify the patient populations most likely to benefit.

## Data Availability

The original contributions presented in the study are included in the article/supplementary material. Further inquiries can be directed to the corresponding author.
